# Sleeping Sickness

**Published:** 1919-04

**Authors:** 


### MEDICAL MISCELLANY

### Sleeping Sickness.

Sleeping sickness is assuming alarming proportions throughout the country and is reported from practically every section. The newspaper reports indicate that it is becoming an epidemic of a kind that is going to require the closest investigation on the part of the physicians of the country.

Illinois reports has forty-three cases of "sleeping sickness," thirteen of which center in Chicago, according to the weekly report made public by Dr. C. St. Claire Drake, director of health.

The first death in New York from "sleeping sickness" was reported to the health department March 14th. Erskine W. Martin, a clerk, 35 years of age, became ill, went to sleep a day later and remained in a state of coma until he died. Health Commissioner Copeland, declaring only eighteen cases of the malady had been reported in Europe and three in New York state of which two had been fatal, said there was no cause for alarm.

Two deaths attributed to lethargic encephalitis were reported by physicians in Boston and Brockton, March 17th. Mrs. Michael S. Russo, who had been asleep for nine days, died in Boston. The death of a child 4 years old from the same cause was announced in Brockton.

Mrs. Thomas De Witte of Murphysboro, Illinois, who slept continuously for three weeks, has given birth to a boy weighing ten pounds. Immediately after the child was born Mrs. De Witte relapsed into sound slumber and several physicians have admitted their inability to awaken her.

The first case of sleeping sickness in Oklahoma was reported Muskogee, March 21st, by Dr. J. T. Nichols. George W. Moats, 30, sank into a stupor at 5 o'clock Thursday afternoon and is still in a comatose state. Efforts to rouse him are only momentarily successful. Moats had influenza six weeks ago. Dizziness and double vision developed a week ago, followed by inability to concentrate his mind, which continued until the stupor set in. Dr. Nichols says the disease is similar in its symptoms to infantile paralysis and he believes it is not contagious.

The first death report in Oklahoma from sleeping sickness occurred March 24th in Durant, when little Dorothy Slaughter died. The little girl was one year of age. She had been asleep about two weeks, and could only be aroused long enough to take nourishment and then lapse back into a slumber. The child lost its eyesight, hearing and also became dumb. One of the peculiar things is the fact that the little child did not have the flu, which all the other sleeping cases in Oklahoma have had.

With announcement of three cases of sleeping sickness, or lethargic encephalitic in New Orleans, March 27th, and eleven in the State, Dr. Oscar Dowling, president of the State Health Board, declared that at the next meeting of the State board an ordinance would be promulgated requiring physicians under the law to report these cases. Cases reported outside of New Orleans are Shreveport one, Richland Parish two, Arnaudville one, Livingston one, Keanerette one, Colfax one and Thibodeaux one.

A number of cases are reported in Texas. Clark Wright, a former army lieutenant, has awakened from a four weeks' sleep at Fort Worth. He knew everything that happened about him, but was unable to arouse himself. Fort Worth physicians who studied the case attribute it either to influenza or to overwork. Beaumont reports one case, a fourteen-year-old boy whose home is eight miles north of the city. He eats heartily, knows all that goes on around and at times laughs at what he thinks his dreams. He slowly recovered after a month of the illness. G. R. West, a farmer near Ballinger was another victim, who could only be aroused to eat, He suffered no unusual pain. John Newman, a farmer aged 79, near Moody is reported slowly recovering after an illness of twenty-one days. An injection of glucose solution was used in this case. Catherine Glittharo, an eighteen-year-old Columbus girl, has been discharged from Santa Rosa Hospital, San Antonio, after six weeks of the sickness. The Brenham Banner-Press reports the case of a negress near Brenham in a comatose condition with no signs of awakening. Mrs. W. H. Childers at Orange, has recovered after a week's illness. The State Health Department on April 9th, reported a death at Merle, Burleson county. Fifteen cases had been reported up to that date of which four had died, one had recovered and no final reports had been made on the others. The death rate is from 30 to 50 per cent.

At the close of March 183 cases had been reported to Washington, though this does not represent the extent of the disease, as reports were then only beginning to come in.

The cases of what is called "sleeping sickness" reported in

### SHERMAN'S
### Bacterial Vaccines
### Efficient - Dependable

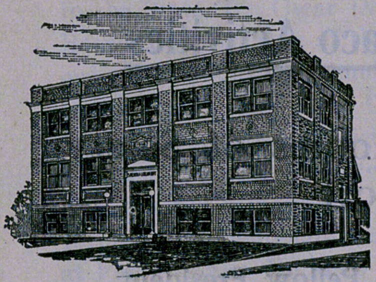

At this season of the year may we call your attention to the ever increasing use of Sherman's Vaccines in the prophylaxis and treatment of HAY FEVER.

DAILY USERS OF VACCINES USE SHERMAN'S

G. H. SHERMAN, M.D.
Detroit, Mich.
U.S.A.

WRITE FOR LITERATURE

Texas differ widely from the disease known by that name in Africa, in the judgment of Dr. D. L. Mumpower, expressed in a letter to Dallas friends. Dr. Mumpower, who is a medical missionary in Africa, and who is now in America to assist in the Methodist Centenary drive for $35,000,000, visited his brother E. V. Mumpower, in Dallas in March. He is familiar with "sleeping sickness" in Africa, and while in Texas he investigated some of the cases reported in this State.

"In Africa sleeping sickness is caused by the bite of a fly," said Dr. Mumpower. "At first the victim appears to have malaria, and within four or five months becomes drowsy, and progressively sleeps for longer periods. If the disease can not be broken up before it reaches this period, it usually proves fatal.

"The disease called sleeping sickness in America is not alarming. It lasts only a few weeks and the patient nearly always recovers, and, often is stronger and better for the experience. Here the disease appears to be rather a form of nervous prostration. But the two sleeping sicknesses are not in the least alike in their symptoms or development."

